# Organophosphate poisoning – shadow-boxing in the intensive care unit

**DOI:** 10.7196/AJTC-CM.2019.v25i3.025

**Published:** 2019-09-17

**Authors:** Richard N van Zyl-Smit, Guy Richards

**Affiliations:** 1 Division of Pulmonology, Groote Schuur Hospital, Cape Town, South Africa; 2 University of the Witwatersrand, Johannesburg, South Africa

## Editorial

 Organophosphate poisoning is not a new phenomenon in South Africa (SA) and is seen regularly in the intensive care unit (ICU), with several reports dating from the mid-1980s.^[1–3]^ Currently, however, organophosphate poisoning appears to be reaching epidemic proportions, with new cases being admitted almost weekly to most public hospitals in the country. These patients provide a challenge to the clinician and the intensivist: the pattern of disease and the variable neurological fallout suggest that this incidence differs from the cases that used to occur following agricultural exposure or intentional overdose. 

 In this issue of the *AJTCCM*, researchers from Chris Hani Baragwanath hospital present a 3-year perspective of cases from 2013 to 2015.^[4]^ During this period, the primary toxin was identified as aldicarb, a carbamate that was originally manufactured by Bayer but which appears now to be illegally imported into SA from across our borders, mostly from Zimbabwe. Bayer had said that they would voluntarily phase out production of aldicarb by 31 December 2014 and that all remaining aldicarb uses would end no later than August 2018.^[4]^ Carbamates differ from organophosphates in that they reversibly inhibit cholinesterases and as such, in contrast to the organophosphates, prolonged paralysis is unlikely to ensue. Length of stay and length of mechanical ventilation are commensurately lower, provided that the paralysis has not resulted in a respiratory arrest prior to admission, as this obviously compromises outcome. As a consequence of the above, organophosphate poisoning has a significantly increased mortality and, in addition, there are frequently other sequelae such as a potentially irreversible axonal neuropathy. 

 In SA, carbamates are sold on the street as a black powder, ostensibly as rat poison. Whereas the compound was originally pure aldicarb, over the period from 2015 to 2018 when poisoning became increasingly common, the composition changed considerably; most now contain a mixture of carbamates and organophosphates and sometimes amitraz (particularly in KwaZulu-Natal Province, and which causes a profound central neurological deficit). Consequently, mortality has considerably increased. 

 These preparations are not commercially prepared and are sold in plastic bags (‘bankies’) without any listing of contents or a package insert. The diagnosis is generally made by the constellation of parasympathetic manifestations and a cholinesterase level, preferably red cell rather than serum cholinesterase, as this gives a better idea of the tissue levels of the toxin. Anecdotally, meiosis and bradycardia are less reliable signs. In Johannesburg, a spectrophotometric method of identification of the ingredients has been developed and is the subject of a PhD thesis; this will be of great value in the future. 

 The dilemma in the ICU at this time is how to predict outcomes when the primary toxin or toxins are not known, and thus the likely length of stay and outcome are less easy to predict. The current study^[4]^ has attempted to evaluate the APACHE II score in their patients. Some studies have evaluated scoring systems, as described in the paper, and have concluded that these scores are valuable; this finding is understandable in that those with paralysis only would have low APACHE II and SOFA scores, but those who had arrested or had some other reason for organ dysfunction would do worse. Although it may be reasonable to try to triage patients in resourcelimited circumstances, we prefer to admit all of these patients and evaluate the responses, given that the specific cognitive effects of the drug itself may transiently affect variables such as the Glasgow Coma Scale. 

 Currently in the ICU we are effectively shadow-boxing. We are treating a very common poisoning syndrome that most frequently follows a suicide attempt, but may also occur following ingestion of an alternative medication or even a homicide attempt, without clear knowledge of the toxin involved and without an adequate means of evaluating outcomes. Tighter regulation of the import, sale and distribution of all insecticides, but particularly the organophosphates and carbamates, is mandatory. Those products which are allowed should have clear labelling and a package insert, and be manufactured according to appropriate industrial standards. These poisonings are epidemic in our society. More attention must be paid to the distribution of organophosphates and, importantly, to limiting individual access to them. Such policies might also allow a shorter and more effective response time for admissions to the ICU. 
